# Efficacy and Safety of Taeeumjowi-tang in Obese Korean Adults: A Double-Blind, Randomized, and Placebo-Controlled Pilot Trial

**DOI:** 10.1155/2013/498935

**Published:** 2013-08-26

**Authors:** Sunju Park, Won Nahmkoong, ChunHoo Cheon, Jeong-Su Park, Bo-Hyoung Jang, Yongcheol Shin, Kyung-Soo Kim, Hoyeon Go, Yun-Kyung Song, Seong-Gyu Ko

**Affiliations:** ^1^Department of Preventive Medicine, College of Korean Medicine, Daejeon University, 62 Daehak-ro, Daejeon 300-716, Republic of Korea; ^2^Department of Preventive Medicine, College of Korean Medicine, Kyung Hee University, 1 Hoegi-dong, Seoul 130-701, Republic of Korea; ^3^Center for Clinical Research & Drug Development, Kyung Hee University, 1 Hoegi-dong, Seoul 130-701, Republic of Korea; ^4^Family Medicine & Integrative Medicine, Seoul St. Mary's Hospital, Catholic University Medical Center, Seoul 137-701, Republic of Korea; ^5^Department of Internal Medicine, College of Korean Medicine, Semyung University, Bongbang-dong, Chungju 380-960, Republic of Korea; ^6^Department of Korean Rehabilitation Medicine, College of Korean Medicine, Gachon University, Seongnam 461-701, Republic of Korea

## Abstract

*Objective*. The purpose of this study was to assess the efficacy and safety of Taeeumjowi-tang (TJ001) as well as to estimate obesity-related factors. *Methods*. This was a 12-week trial with 5 visits. A total of 102 participants of both genders were randomized to either TJ001 (*n* = 57) group or the placebo group (*n* = 55). Subjects were administered 7 g of either TJ001 or placebo 3 times a day. The primary outcome was a rate of subjects who lost 5% or more of initial weight. Secondary outcomes included anthropometric parameters, lipid profiles, and body fat composition. *Results*. The subject response rate of ≥5% weight loss compared to baseline was similar in both groups, and no statistically significant difference was observed (*P* = 0.87). Changes in anthropometric parameters were greater during the first 4 weeks in the treatment group (*P* < 0.0001). There were no significant changes in both within groups and between groups for lipid profile and body fat composition. No adverse event was reported in either group. *Conclusion*. Although the difference between the groups regarding a rate of subjects who lost 5% or more of initial weight did not show statistical significance, TJ001 appears to be beneficial in safely controlling weight.

## 1. Introduction

Obesity is defined as abnormal or excessive fat accumulation that threatens health. Obesity not only leads to physical and external complications but also causes various diseases, such as cardiovascular disease, musculoskeletal disease, endocrinopathy, and psychological disease [1]. The World Health Organization (WHO) considers obesity to be lifestyle-related disease and calls for its management. A high prevalence of obesity has become a global problem. If the current trend continues, the obese population will be 700 million in 2015 and one-third of the world population in 2025. The most recent Korean National Health and Nutrition Examination Survey (KNHANES) found that 35.2% of male adults, 28.6% of female adults, and 31.9% of adults aged over 19 were overweight according to BMI calculations [2]. Westernized eating habits, such as an increased intake of fat, salt, and sugar, have played a large part in the increasing number of obese individuals in Korea.

Socioeconomic costs for obesity treatment in Korea were estimated to be approximately 205 billion won to 422.5 billion won in 1998 [3], and in 2005, this treatment incurred direct expenses of 177 billion won, an overhead cost of 715.2 billion won, and a total cost of 1792.2 billion won, four times the cost in 1998. The trend will continue with medical cost inflation and with the increasing number of obese individuals. 

Over a period of many years, numerous medications have been developed and used to treat obesity. However, most of the drugs have been withdrawn because of serious adverse effects [4]. Recently, sibutramine was withdrawn due to an association with increased cardiovascular disease risk [5]. In this context, a demand for novel treatments exists, and herbal medicines have received considerable attention due to the perception that they have no adverse effects.

Taeeumjowi-tang (TJ001) is a traditional Korean medicine preparation for Tae-eumin exterior cold symptoms. It is recorded in I Je-ma's Donguisusebowon, which is considered to be the bible of Sasang constitutional medicine theory. Taeeumjowi-tang works as a treatment for dyspepsia, stuffiness and the sensation of fullness, diarrhea, and wind stroke [1, 6]. Taeeumjowi-tang tonifies the exhale-dispersing strength of the lung and represses the inhale-gathering strength of the liver. It is used to treat obesity by inducing weight loss through metabolic activation. Animal studies and case reports have already demonstrated weight loss success and the anorectic effect of Taeeumjowi-tang [7, 8]. Based on these research results, Taeeumjowi-tang has been suggested as an alternative treatment for obesity. The aims of the present study were to examine estimates of obesity-related variables, to obtain data to suggest an appropriate primary endpoint and treatment period, and to assess the clinical efficacy and safety of Taeeumjowi-tang as a viable treatment for obesity. 

## 2. Methods

### 2.1. Study Design and Settings

This study was a 12-week randomised, double-blind, and placebo-controlled clinical trial conducted at four tertiary university hospitals in South Korea. The study was approved by the institutional review board (IRB) at the Catholic University of Korea Seoul St. Mary's Hospital, Dongguk University Ilsan Oriental Hospital, Semyung University Oriental Medicine Hospital, and Kyungwon Gil Oriental Medical Hospital. Subjects were randomized by a web-based randomization program that was developed by an independent biostatistician coded with a block size of 4. The program was designed to ensure concealment of the sequence as well as allocation to the treatment and placebo groups in a 1 : 1 ratio for each site. The research coordinator or the study staff assigned the subjects after the randomization, and the pharmacist provided the interventions to the subjects according to the randomization number. All study participants including the subjects were blinded to the group assignment. A sealed envelope copy of the code was kept to CRO in case of emergency and for the analysis. The study was conducted from December 2009 to June 2012, and the trial was registered in Current Controlled Trials (ISRCTN87153759) [9].

### 2.2. Subjects

The subjects were recruited by advertising on the bulletin board of each hospital. Written informed consent was obtained from each subject prior to participation. The subjects were obese Koreans of both genders with an age range of 18 to 65 years. In this trial, 102 subjects with BMI ≥ 30 kg/m^2^ or BMI 27 to 30 kg/m^2^ with properly controlled hypertension, noninsulin-dependent diabetes mellitus of fasting blood glucose <7.8 mmol/L (140 mg/dL), properly treated hyperlipidemia, ≥236 mg/dL total cholesterol, or ≥150 mg/dL triglycerides at the screening stage were included. The exclusion criteria consisted of the following: (1) endocrine disease except for type 2 diabetes mellitus; (2) heart diseases; (3) uncontrolled hypertension; (4) malignant tumor; (5) severe renal and liver disability; (6) history or existence of neurological or psychological disease including eating disorder; (7) use of medication affecting weight and blood pressure within the past 3 months; (8) forbidden treatments; (9) renal or hepatic disease; (10) inability to follow instructions; (11) pregnant, planning a pregnancy but not in agreement with proper contraception or lactating women. 

### 2.3. Study Schedule

The study was conducted within a 1-week screening period followed by a 12-week treatment period with a total of 5 visits. Baseline measurements were carried out during the 1-week screening phase. After satisfying the inclusion criteria, the subjects were equally randomized into either the TJ001 group or the placebo group. Physical examinations (body weight, BMI, and waist and hip circumferences, waist and hip ratio (WHR)) were taken at every visit, throughout the 12-week period for the efficacy assessment. Lipid profiles (total cholesterol, low density lipoprotein cholesterol (LDL) cholesterol, and triglycerides), body fat composition (total fat area, visceral fat area, and subcutaneous fat area), C-reactive protein, and self-assessment questionnaires were measured at the 2nd and 5th visits. 

Safety parameters were assessed by measuring changes in vital signs, general physical examinations, blood and urine test results, and self-reported symptoms. 

The subjects were asked to record their diet in dietary planners and to maintain s hypocaloric diet (1,500 kcal/day for men and 1,200 kcal/day for women) during the course of the trial. Subjects were instructed to maintain their usual exercise but not to intensify their activities. Life style management factors, such as dietary intake and exercise, were recorded and counseled at every visit but were not strictly controlled. 

Subjects received either 7 g of TJ001 or placebo extracts three times daily for 12 weeks. Both TJ001 and placebo extracts were provided by HANPOONG Pharm & Foods Co. Ltd (Jeonju-si, South Korea) produced by Good Manufacturing Practice (GMP) facilities. The ingredients of TJ001 were *Semen Coicis* 3.75 g, *Semen Castaneae* 3.75 g, *Semen Raphani* 2.5 g, *Schisandrae Fructus* 1.25 g, *Liriopis tuber* 1.25 g, *Herba Ephedrae* 1.25 g, *Radix platycodi* 1.25 g, and *Acori Tatarinowii Rhizoma* 1.25 g. The placebo granules were identical to TJ001 in appearance, color, smell, and taste. 

### 2.4. Screening

Body weight, height, waist and hip circumference, demographic characteristics, measurements of vital signs, medical and drug use history, smoking and drinking status, general physical examination, laboratory tests, electrocardiography, and pregnancy tests were included in the screening. Blood samples were analyzed at the central laboratory (Eone Reference Laboratory, Seoul, Korea).

### 2.5. Efficacy

The primary outcome was the rate of subjects who lost 5% or more compared with baseline body weight. The secondary outcomes were changes in body weight (kg), body mass index (BMI, kg/m^2^), waist circumference (WC, cm), hip circumference (HC, cm), waist/hip circumference ratio (WHR), blood pressure (mmHg), lipid profile (total cholesterol (TC, mg/dL), HDL cholesterol (mg/dL), LDL cholesterol (mg/dL), and triglyceride (TG, mg/dL) levels. In addition, body fat composition such as total fat area (TFA, cm^2^), visceral fat area (VFA, cm^2^), and subcutaneous fat area (SFA, cm^2^) was assessed by abdominal computed tomography. Subject body weight was measured in light clothing and without shoes. Weight and height were measured by a balance scale with a movable headpiece rod in the standing position to the nearest 0.1 cm and 0.1 kg, respectively. BMI was estimated by dividing weight in kilograms by height in square meters. Both WC and HC were recorded to the nearest 0.1 cm with a plastic tape. The WC was measured at the suprailiac line according to NIH protocol [10], and the HC was measured at the horizontal level of the largest part of the buttocks. Laboratory data (fasting plasma glucose concentration (mg/dL) and C-reactive protein (CRP, mg/L) were also included in the secondary outcomes. Korean Obesity-related Quality of Life (KOQOL) and Korean version of Eating Attitudes Test-26 (KEAT-26) questionnaires scores were evaluated. All measurements were taken by well-trained medical staffs by standard operating procedure (SOP).

### 2.6. Safety

Vital signs such as resting blood pressure, general physical examinations, and laboratory data (AST, ALT, BUN, and creatinine) were measured at every visit for the safety assessments. Adverse events were reported to the case report form regardless of their association with the intervention.

### 2.7. Statistical Analysis

All statistical analyses were done to achieve a statistical power of 80%, and a *P* value of <0.05 was considered statistically significant. The sample size was calculated based on the null hypothesis that the rate of subjects who lost 5% or greater of the baseline weight, that is, the primary endpoint in the TJ001 group, would be higher than that of the placebo group. As pilot studies have not been conducted for TJ001, the 5% or greater responder rate of the baseline weight in each group was set based on the other reference literature [11]. Allowing for a 20% dropout rate, the minimum sample size of 104 subjects with 52 subjects per group was estimated for the study. 

Both efficacy and safety outcomes were analyzed by an intention-to-treat (ITT) analysis. All randomized subjects with at least one visit after the administration of the interventions were included in the ITT data set. Missing data were handled by the last-value-carried-forward (LOCF) method. Continuous variables were described as the mean ± SD, and categorical variable such as gender was presented as percentages. The normal distribution assumption was checked before the analysis was conducted. Data were log transformed when necessary. 

As all data satisfied the normality assumption, the baseline characteristics of the efficacy and safety outcomes were compared by either Student's *t*-test for continuous variables or by the chi-square test (Fisher's exact test if the expected value was less than 5) for categorical data. 

Efficacy parameters and vital signs were compared at every visit for analysis between groups. Student's *t*-test was applied to assess the difference between groups, while the paired *t*-test was used to examine differences within groups. Repeated measures ANOVA was used to assess time by group interaction in repeated measurements. ANCOVA was used to evaluate all efficacy outcomes to determine whether the between groups were significantly different at the end of the study using baseline measures as a covariate.

Data were analyzed using the SPSS Statistic software, version 19.0 for Windows (SPSS, Chicago, IL).

## 3. Results 

### 3.1. Participant Flow

**Figure 1:**
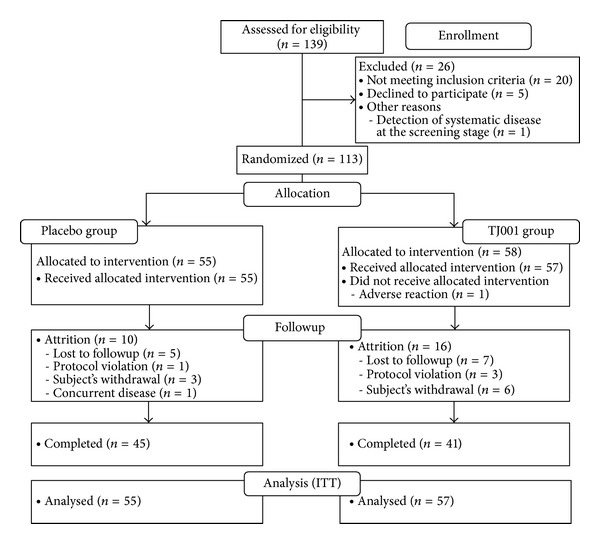
Disposition of the subjects in TJ001 trial.

Participant flow is shown in Figure 1. A total of 139 participants were assessed for eligibility, 21 were disqualified, and 5 decided not to participate. One hundred and thirteen subjects were randomized, 58 to the treatment group and 55 to the placebo group. As one participant in the treatment group had allergic dermatitis after randomization, this subject was excluded from the ITT data set before receiving the intervention. Thus, 55 individuals treated with placebo and 57 treated with TJ001 were included in the ITT analyses. After 12 weeks, 45 completed the trial in the placebo group and 41 in the treatment group. 

The baseline characteristics of the trial participants are described in Table 1. Subjects between groups were not different initially except for total cholesterol (*P* = 0.0180). Distribution of gender was also not significantly different between groups (*P* = 0.3842). 

### 3.2. Efficacy Analysis

#### 3.2.1. Primary Outcome

**Figure 2:**
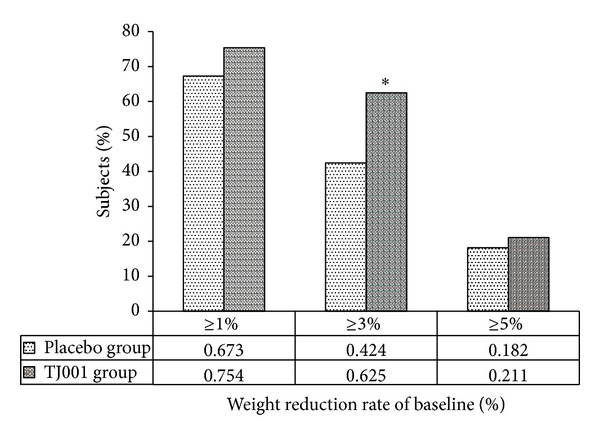
Percentage of subjects who had 1%, 3%, and 5% or more weight reduction (*: statistically significant between groups, *P* < 0.05).

After the 12-week treatment period, the rate of subjects who lost at least 5% of baseline was similar in both groups with no statistically significant difference (TJ001 group and placebo group, 21.1% and 18.2%, resp., *P* = 0.87, Figure 2). However, the subject rates of at least 3% weight loss were significantly higher in the treatment group compared with the placebo group (*P* = 0.01, Figure 2).

#### 3.2.2. Secondary Outcomes: Anthropometric Measurements

**Figure 3:**
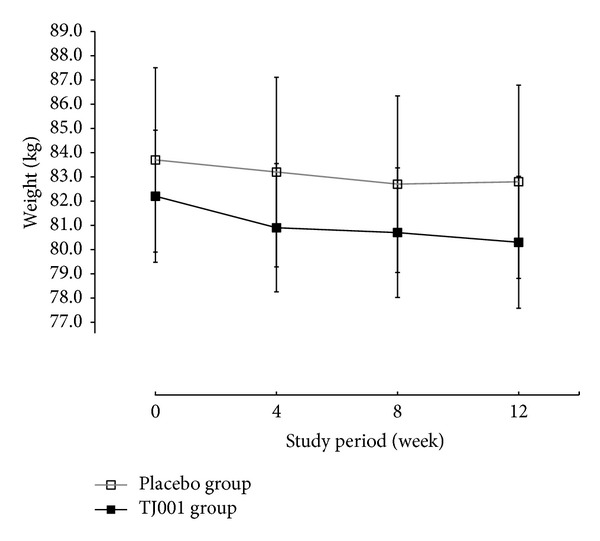
Changes in average body weight over time. Data are represented as the mean ± S.D. of weight at each week. The full squares with a solid line indicate the herbal treatment (TJ001) group, and the empty squares with a grey line depict the placebo group. No statistically significant differences were found between the groups at each week.

The mean weights at each time point are shown in Figure 3. The change patterns in BMI, WC, and HC paralleled those in weight change in the two groups (not shown). Weight, BMI, WC, and HC reductions for every 4th week were greater during the first 4 weeks in the treatment group compared with the other treatment period, showing a plateau between the 4th and 8th weeks. This pattern was more definite in the treatment group than in the placebo group. In contrast, the weight loss in the placebo group did not proceed after week 8. 

A significant decrease in weight was observed in both groups but was greater in the treatment group (−1.95 ± 3.35 kg, *P* < 0.0001); however, it was not significant between groups (Table 2). The within-group changes in BMI, WC, and HC were also significant in each group but not significant for the between-group comparison. For WHR, the within-group changes were significant only in the treatment group (*P* = 0.0108). Repeated measures ANOVA results depicted that there were no significant visit and group interaction (time × treatment, Table 2).

#### 3.2.3. Secondary Outcomes: Lipid Profile, Body Fat Composition, and CRP

There were no significant changes both within groups and between groups in regards to lipid profile and body fat composition (Table 3). Questionnaire scores were also not statistically significant for both the within-group and between-group assessments (not shown). 

The distribution of Sasang constitution according to the Questionnaire for the Sasang Constitution Classification (QSCC) II in this study was as follows: 82 Tae-eum types, 2 So-yang types, 1 So-eum type, 1 Tae-yang type, and 20 unclassified subjects.

### 3.3. Safety Analyses

**Figure 4:**
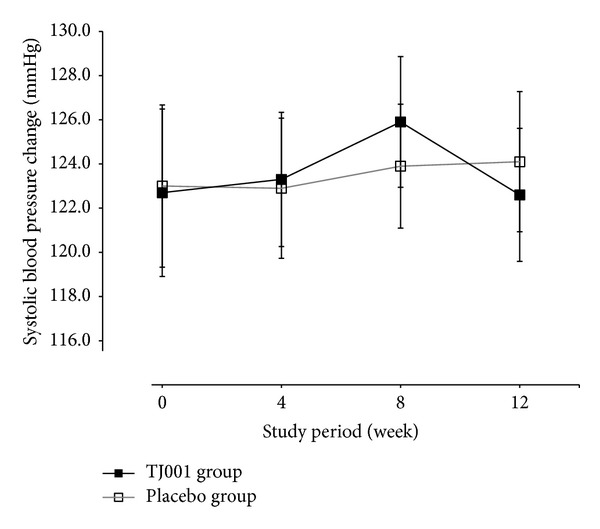
Changes in average systolic blood pressure (SBP) over time. Data are shown as mean ± S.D. of SBP at each week. Full squares with a solid line indicate the herbal treatment (TJ001) group, and empty squares with a grey line depict the placebo group. No statistically significant differences were found between groups at each week.

Mean systolic and diastolic blood pressure and creatinine were not different between groups at any week nor were they different from the baseline measurements within each group at the final visit of the study (Table 4). Systolic blood pressure increased from baseline to week 8 (Figure 4). However, after the 8th week, systolic blood pressure decreased only in the treatment group. There was a significant change in pulse observed in the placebo group and in the between-group comparison (*P* = 0.0058 and *P* = 0.018, resp.). 

Although changes in AST in either group (placebo group and treatment group *P* = 0.0315 and 0.0003, resp.) and in ALT in the treatment group were statistically significant (*P* = 0.0038) from baseline, they were not in the abnormal range. All variables of subjects in both groups were in the clinically normal range at end of the study.

### 3.4. Adverse Effects

One subject in the treatment group developed allergic dermatitis before the administration of the intervention. Therefore this can be ruled out as a potential treatment-related adverse effect. In this trial, no adverse effects were reported. 

### 3.5. Compliance

Overall compliance of treatment was 24% with 70.7% in the TJ001 group and 81.8% in the placebo group.

## 4. Discussion 

Up-to-date, safety, and efficacy matters are the main issues for human weight controlling agents [12–14]. Among the many efforts to evaluate the efficacy and safety of herbal antiobesity agents [15, 16], this pilot study is the first randomized clinical trial for Taeeumjowi-tang in the Korean population. 

Because the beneficial effect of at least a 5% weight reduction of baseline has been previously studied [17], we set the rate of subjects who lost 5% or more of baseline weight for primary endpoint.

In this trial, groups did not achieved ≥5% weight reduction (18.2% and 21.1% in the placebo group and the TJ001 group, resp.). The difference between groups was only 2.9%, which was not statistically significant (Figure 2). The weight loss in both groups might be due to the large quantity of the extracts that result in a decrease in food intake. This result indicates that weight loss can be more efficient when accompanied with a strict diet and exercise counseling. However, a 3% or greater reduction rate from baseline weight is statistically significant between groups, suggesting that the appropriate primary endpoint for herbal preparation might be between a 3 and 5% reduction rates of initial weight. However, reduction patterns were different in each group (Figure 3). The weight, WC, and HC reductions were greater during the first 4 weeks in the treatment group, with a plateau between the 4th and 8th week and a subsequent decrease after 8 weeks. In contrast, the plateau was seen after 8 weeks in the placebo group. This pattern indicates that the weight reducing effect of TJ001 might be beneficial for relatively short-term or long-term use. Therefore, the appropriate treatment period of TJ001 would be less than 4 weeks or more than 12 weeks (long term). 

A total of 62.5% subjects in the treatment group reduced at least 3% of their initial weight after 12 weeks, compared with those in the placebo group (42.4%, Figure 2), and the difference between the two groups was found to be statistically significant (*P* = 0.015). These results suggest that ≥3% weight loss might be an adequate primary endpoint for herbal preparation for less than 12 weeks. In Korean Medicine, obesity originates from the accumulation of body wastes and the congestion of qi. While the mechanism of actions of Taeeumjowi-tang is not fully understood, components of Taeeumjowi-tang and circulating effects, such as fat oxidation and thermogenic action, might promote weight reduction and other responses, such as a decrease in waist and hip circumference [12].

Ideally, any weight reducing agent should promote the loss of body fat as well as body weight. However, regardless of the reduction trend in the anthropometric parameters, the differences in lipid profile and body fat composition both within and between groups were not statistically significant (Table 3). The Korean Obesity-related Quality of Life (KOQOL) and Korean version of Eating Attitudes Test-26 (KEAT-26) questionnaires also showed no statistical significance in both within and between groups. Additionally, the Sasang distribution results suggest that obesity is diagnosed predominantly in Tae-eum types (*n* = 82, 77.4%), which support the Sasang constitutional medicine theory that obesity prevalence is higher in Tae-eum types than in the other three constitutional types.

For safety-related parameters, although some parameters such as AST and ALT were significant within the treatment group, all observed changes in the variables were in the clinically normal range. No significant adverse effects were reported in the present study. Some participants withdrew themselves because of personal reasons but not due to treatment-related side effects.

The limitations of this study are as follows. First, as previously described, the trial included both genders, which might be a cause of heterogeneity, as the gender difference in weight loss pattern and magnitude are well known [18]. However, we could not perform subgroup analysis for gender because the number of male subjects was too small (Table 1). Second, a TJ001 efficacy assessment longer than 12 weeks could not be performed because of the limited study budget. Therefore, as these are relatively short-term results, it is unclear as to whether weight loss can be maintained, that is, no trajectory weight gain. 

Third, the drop-out rate was exceeded in this study (Figure 1). The main reason for the high drop-out rate was lost to followup due to difficulty in managing subjects. The medical staff mostly covered role of the clinical research coordinator (CRC). This implies that the management of subjects with sufficient clinical research staffs is one of the major factors for controlling the drop-out rate.

Finally, the study was performed without strict lifestyle management, which might affect weight control. To identify true intervention effects, other variables, such as diet and exercise, should be controlled. 

The strengths of the study include the implication that TJ001 is an effective treatment for weight control in the Korean population and that the results of this trial can be extended to other weight loss studies based on the measurements of obesity-related variables. 

## 5. Conclusion

In conclusion, despite the several limitations and lack of statistical significance between the groups, the present study identified the possibility of and trend for the safe weight reducing effect of Taeeumjowi-tang. Additionally, results from this study will offer preliminary data for large-scale and precise antiobesity trials in the Korean population. Furthermore, the efficacy and safety of relatively long-term use needs to be validated. 

## Figures and Tables

**Table 1:** Baseline characteristics of TJ001 trial subjects.

	Group		*P* value*
	Placebo group (*N* = 55)	TJ001 group (*N* = 57)	
	mean ± S.D.	mean ± S.D.	
Age, year	38.8 ± 10.1	39.2 ± 9.5	0.8475
Anthropometric measurements			
Height, cm	161.8 ± 9.2	160.8 ± 6.4	0.4998
Weight, kg	83.7 ± 14.4	82.2 ± 10.5	0.5458
BMI, kg/m^2^	31.9 ± 3.8	31.8 ± 2.6	0.8598
Waist circumference, cm	100.4 ± 9.5	99.6 ± 7.0	0.6259
Hip circumference, cm	110.4 ± 8.8	109.9 ± 6.0	0.7096
Waist-to-hip ratio (WHR)	0.91 ± 0.05	0.90 ± 0.05	0.6934
Lipid profile			
Cholesterol			
Total cholesterol, mg/dL	207.3 ± 36.8	191.7 ± 31.5	0.0180
LDL cholesterol, mg/dL	160.39 ± 36.57	146.43 ± 30.37	0.0300
HDL cholesterol, mg/dL	46.9 ± 9.9	46.1 ± 10.6	0.7161
Triglyceride, mg/dL	152.2 ± 87.2	134.6 ± 66.9	0.2352
Abdominal computed tomography (CT)			
Total fat area (TFA), cm^2^	402.8 ± 153.9	392.5 ± 134.6	0.7676**
Visceral fat area (VFA), cm^2^	118.2 ± 58.1	110.1 ± 47	0.5073**
Subcutaneous fat area (SFA), cm^2^	283.6 ± 116.3	282.1 ± 103	0.9149**
VFA/SFA	0.44 ± 0.21	0.40 ± 0.17	0.2524
C-reactive protein (CRP), mg/dL	0.19 ± 0.24	0.22 ± 0.36	0.6786**
Questionnaires			
KOQOL	33.7 ± 8.2	33.5 ± 6.8	0.8815
KEAT-26	13.5 ± 5.5	12.1 ± 4.8	0.1665
Energy intake, kcal/day	1859 ± 635.9	1823.1 ± 623	0.7671
Pulse, bpm	76.9 ± 9.3	75.3 ± 7.4	0.3072
Systolic blood pressure (SBP), mmHg	123 ± 13.9	122.7 ± 14.6	0.9175
Diastolic blood pressure (DBP), mmHg	77.8 ± 10.8	76.6 ± 9.2	0.5433
AST, IU/L	24.09 ± 10.97	27.86 ± 16.16	0.1395**
ALT, IU/L	30.22 ± 23.55	28.91 ± 18.92	0.9326**
BUN, mg/dL	13.39 ± 2.89	14.45 ± 10.42	0.8895**
Creatinine, mg/dL	0.83 ± 0.17	0.78 ± 0.18	0.1415**

Number of subjects (*n*)	*n* (%)	*n* (%)	*P* value^†^

Gender			
Male, *n* (%)	10 (18.2%)	7 (12.3%)	0.3842
Female, *n* (%)	45 (81.8%)	50 (87.7%)	

S.D.: standard deviation; BMI: body mass index; LDL: low-density lipoprotein; HDL: high-density lipoprotein CT: computed tomography; KOQOL: Korean Obesity-related Quality of Life, KEAT-26: Korean version of Eating Attitudes Test-26; BUN: blood urea nitrogen; AST: aspartate aminotransferase; ALT: Alanine transaminase. *Continuous variables were analyzed by independent samples *t*-test. **P values after log transformed, as they did not satisfy normality assumption. ^†^Categorical variables were analyzed by chi-square test. *P* < 0.05: considered statistically significant.

**Table 2:** Analyses of anthropometric parameters.

Secondary outcomes (Anthropometric parameters)	Period	Group				P*	(*P*_inter_)
		Placebo group (*N* = 55)		TJ001 group (*N* = 57)			
		Mean ± S.D.	(*P*_intra_)	Mean ± S.D.	(*P*_intra_)		
Weight, kg	Baseline	83.7 ± 14.4	—	82.2 ± 10.5	—	0.5458	—
	week 4	83.2 ± 14.8		80.9 ± 10.2		0.3272	
	week 8	82.7 ± 13.8		80.7 ± 10.3		0.3900	
	week 12	82.8 ± 15.1		80.3 ± 10.5		0.3080	
	Δ0–12	−0.88 ± 3.05	0.0371	−1.95 ± 3.35	<0.0001	—	0.0708
	ANOVA	visit X group interaction: *P* = 0.0816					
Body mass index (BMI), kg/cm^2^	Baseline	31.9 ± 3.80	—	31.8 ± 2.60	—	0.8598	—
	week 4	31.6 ± 3.90		31.2 ± 2.60		0.5660	
	week 8	31.4 ± 3.90		31.2 ± 2.80		0.7407	
	week 12	31.4 ± 4.10		31.0 ± 2.80		0.5420	
	Δ0–12	−0.45 ± 1.17	0.0061	−0.75 ± 1.27	<0.0001	—	0.1680
	ANOVA	visit X group interaction: *P* = 0.3703					
Waist circumference (WC), cm	Baseline	100.4 ± 9.50	—	99.6 ± 7.00	—	0.6259	—
	week 4	99.7 ± 9.60		97.6 ± 7.40		0.1932	
	week 8	98.6 ± 9.30		97.3 ± 7.70		0.3938	
	week 12	97.3 ± 11.70		96.3 ± 7.90		0.6056	
	Δ0–12	−3.09 ± 8.57	0.010	−3.29 ± 4.54	<0.0001	—	0.7665
	ANOVA	visit X group interaction: *P* = 0.4725					
Hip circumference (HC), cm	Baseline	110.4 ± 8.80	—	109.9 ± 6.00	—	0.7096	—
	week 4	109.8 ± 8.30		108.4 ± 5.70		0.3027	
	week 8	109.2 ± 8.40		108.1 ± 5.50		0.4026	
	week 12	108.6 ± 8.30		107.6 ± 5.70		0.4330	
	Δ0–12	−1.82 ± 3.48	0.0003	−2.35 ± 3.51	<0.0001	—	0.3183
	ANOVA	visit X group interaction: *P* = 0.3456					
Waist/hip ratio (WHR)	Baseline	0.91 ± 0.05	—	0.90 ± 0.05	—	0.6934	—
	week 4	0.91 ± 0.05		0.90 ± 0.05		0.4217	
	week 8	0.90 ± 0.04		0.90 ± 0.06		0.6775	
	week 12	0.90 ± 0.05		0.89 ± 0.05		0.4159	
	Δ0–12	−0.0073 ± 0.04	0.1307	−0.0114 ± 0.03	0.0108	—	0.4058
	ANOVA	visit X group interaction: *P* = 0.7758					

P*: A *P* value for between-group comparison at each visit and visit X group interaction using ANOVA.

(*P*_intra_): A *P* value for within-group comparison between baseline and 5th visit by paired *t*-test.

(*P*_inter_): A *P* value for between-group comparison after 12-week treatment using ANCOVA (baseline as covariate).

Δ0–12: Changes between baseline and the 5th visit (after 12-week treatment).

*P* < 0.05 were considered statistically significant.

**Table 3:** Analyses of lipid profile, body fat composition, and CRP.

Secondary outcomes (lipid profile, body fat composition, and CRP)	Period	Group				P*	(*P*_inter_)
		Placebo group (*N* = 55)		TJ001 group (*N* = 57)			
		Mean ± S.D.	(*P*_intra_)	Mean ± S.D.	(*P*_intra_)		
Lipid profile							
Cholesterol							
Total cholesterol, mg/dL	Baseline	207.3 ± 36.8	0.1142	191.7 ± 31.5	0.0983	0.0180	0.8980
	week 12	201.9 ± 39.6		187.2 ± 34.3		0.0374	
HDL cholesterol, mg/dL	Baseline	46.9 ± 9.90	0.8695	46.1 ± 10.6	0.7240	0.7161	0.2717
	week 12	47.2 ± 10.9		45.9 ± 9.70		0.5249	
Triglyceride, g/dL	Baseline	152.2 ± 87.2	0.4709	134.6 ± 66.9	0.8306	0.2352	0.4977
	week 12	143.3 ± 87.9		133.3 ± 64.5		0.4963	
Body fat composition							
Total fat area (TFA), cm^2^	Baseline	402.8 ± 153.9	0.2103	392.5 ± 134.6	0.1201	0.7105	0.5205
	week 12	387.9 ± 134.2		383.4 ± 137.0		0.8601	
Visceral fat area (VFA), cm^2^	Baseline	118.2 ± 58.1	0.4255	110.1 ± 47.0	0.2669	0.4252	0.6621
	week 12	115.9 ± 58.5		105.4 ± 51.8		0.3253	
Subcutaneous fat area (SFA), cm^2^	Baseline	283.6 ± 116.3	0.2898	282.1 ± 103.0	0.2582	0.9425	0.3767
	week 12	272.1 ± 102.1		278.2 ± 105.2		0.7579	
VFA/SFA	Baseline	0.44 ± 0.21	0.3921	0.40 ± 0.17	0.5682	0.2524	0.8109
	week 12	0.43 ± 0.21		0.39 ± 0.19		0.3055	
C-reactive protein (CRP), mg/dL	Baseline	0.83 ± 0.17	0.3766	0.78 ± 0.18	0.6834	0.1547	0.3731
	week 12	0.85 ± 0.19		0.79 ± 0.19		0.0721	

P*: A *P* value for between-group comparison at each visit and visit X group interaction using ANOVA.

(*P*_intra_): A *P* value for within-group comparison between baseline and 5th visit by paired *t*-test.

(*P*_inter_): A *P* value for between-group comparison after 12-week treatment using ANCOVA (baseline as covariate).

*P* < 0.05 were considered statistically significant.

**Table 4:** Safety analyses of TJ001.

Safety parameters	Period	Group				P*	(*P*_inter_)
		Placebo group (*N* = 55)		TJ001 group (*N* = 57)			
		Mean ± S.D.	(*P*_intra_)	Mean ± S.D.	(*P*_intra_)		
Blood pressure							
Systolic, mmHg	Baseline	123.0 ± 13.9	0.5360	122.7 ± 14.6	0.9712	0.9175	0.3607
	week 4	122.9 ± 12.0		123.3 ± 11.7		0.8620	
	week 8	123.9 ± 10.6		125.9 ± 11.4		0.3451	
	week 12	124.1 ± 12.0		122.6 ± 11.6		0.5248	
	ANOVA	visit X group interaction: *P* = 0.4121					
Diastolic, mmHg	Baseline	77.8 ± 10.8	0.9167	76.6 ± 9.20	0.4862	0.5433	0.7461
	week 4	76.2 ± 9.00		77.1 ± 8.10		0.5852	
	week 8	79.7 ± 16.20		78.8 ± 8.20		0.6966	
	week 12	77.9 ± 9.20		77.5 ± 7.60		0.7933	
	ANOVA	visit X group interaction: *P* = 0.75					
Pulse, bpm	Baseline	76.9 ± 9.30	0.0058	75.3 ± 7.40	0.7718	0.3072	0.0184
	week 4	76.3 ± 7.70		76.3 ± 8.00		0.9851	
	week 8	75.5 ± 7.70		76.9 ± 7.60		0.3108	
	week 12	73.6 ± 7.20		75.6 ± 8.50		0.1743	
	ANOVA	visit X group interaction: *P* = 0.0475					
AST, IU/L	Baseline	24.1 ± 11.0	0.0315	27.9 ± 16.2	0.0003	0.1505	0.0544
	week 12	22 ± 8.80		21.9 ± 12.3		0.9798	
ALT, IU/L	Baseline	30.2 ± 23.5	0.1236	28.9 ± 18.9	0.0038	0.7464	0.3573
	week 12	27.3 ± 21.7		22.1 ± 13.5		0.1327	
BUN, mg/dL	Baseline	13.4 ± 2.90	0.1252	14.5 ± 10.4	0.2556	0.4616	0.7000
	week 12	12.8 ± 2.60		12.7 ± 4.10		0.8953	
Creatinine, mg/dL	Baseline	0.83 ± 0.17	0.3766	0.78 ± 0.18	0.6834	0.1547	0.3731
	week 12	0.85 ± 0.19		0.79 ± 0.19		0.0721	

P*: A *P* value for between-group comparison at each visit and visit X group interaction using ANOVA.

(*P*_intra_): A *P* value for within-group comparison between baseline and 5th visit by paired *t*-test.

(*P*_inter_): A *P* value for between-group comparison after 12-week treatment using ANCOVA (baseline as covariate).

*P* < 0.05 were considered statistically significant.
